# 2,2′-[(1*E*,1′*E*)-2,2′-(2,5-Dibut­oxy-1,4-phenyl­ene)bis­(ethene-2,1-di­yl)]dipyridine

**DOI:** 10.1107/S1600536810013656

**Published:** 2010-06-30

**Authors:** Rui-Long Zhang, Zhao-Di Liu, Jie-Ying Wu

**Affiliations:** a Department of Chemistry, Anhui University, Hefei 230039, People’s Republic of China, and Key Laboratory of Functional Inorganic Materials Chemistry, Hefei 230039, People’s Republic of China

## Abstract

The centrosymmetric title mol­ecule, C_28_H_32_N_2_O_2_, has a central benzene ring subsituted in the 1- and 4-positions by (ethene-2,1-di­yl)pyridine groups, and in the 2- and 5-positions by but­oxy groups. The whole mol­ecule is X-shaped and relatively flat, the dihedral angle between the pyridine and the central benzene ring being 11.29 (10)°. In the crystal, neighboring mol­ecules are linked by weak C—H⋯N inter­actions, forming a two-dimensional undulating network.

## Related literature

For information on pyridine-based photo-refractive materials, see: Naumov *et al.* (2002 ▶); Liu *et al.* (2008 ▶).
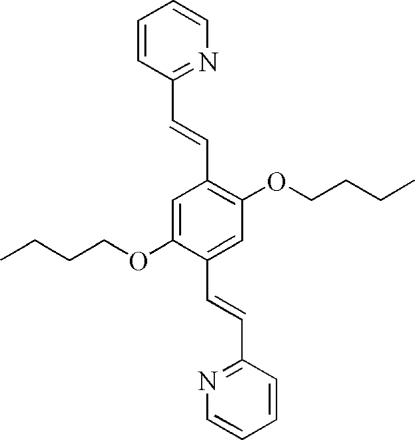


## Experimental

### 

#### Crystal data

C_28_H_32_N_2_O_2_
*M* *_r_* = 428.56
Monoclinic, 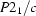

*a* = 8.882 (5) Å
*b* = 13.892 (5) Å
*c* = 10.387 (5) Å
β = 107.392 (5)°
*V* = 1223.0 (10) Å^3^
*Z* = 2
Mo *K*α radiation
μ = 0.07 mm^−1^
*T* = 298 K
0.50 × 0.30 × 0.20 mm

#### Data collection

Bruker SMART CCD area-detector diffractometer
Absorption correction: multi-scan (*SADABS*; Sheldrick, 1996 ▶) *T*_min_ = 0.964, *T*_max_ = 0.986
8512 measured reflections
2162 independent reflections
1385 reflections with *I* > 2σ(*I*)
*R*_int_ = 0.040

#### Refinement

*R*[*F* ^2^ > 2σ(*F* ^2^)] = 0.049
*wR*(*F* ^2^) = 0.154
*S* = 1.03
2162 reflections
146 parameters
H-atom parameters constrained
Δρ_max_ = 0.16 e Å^−3^
Δρ_min_ = −0.16 e Å^−3^

### 

Data collection: *SMART* (Bruker, 2007 ▶); cell refinement: *SAINT* (Bruker, 2007 ▶); data reduction: *SAINT*; program(s) used to solve structure: *SHELXS97* (Sheldrick, 2008 ▶); program(s) used to refine structure: *SHELXL97* (Sheldrick, 2008 ▶); molecular graphics: *DIAMOND* (Brandenburg & Putz, 2008 ▶); software used to prepare material for publication: *SHELXL97*.

## Supplementary Material

Crystal structure: contains datablocks I, global. DOI: 10.1107/S1600536810013656/su2172sup1.cif

Structure factors: contains datablocks I. DOI: 10.1107/S1600536810013656/su2172Isup2.hkl

Additional supplementary materials:  crystallographic information; 3D view; checkCIF report

## Figures and Tables

**Table 1:** Hydrogen-bond geometry (Å, °)

*D*—H⋯*A*	*D*—H	H⋯*A*	*D*⋯*A*	*D*—H⋯*A*
C5—H5⋯N1^i^	0.93	2.70	3.446 (3)	138

Symmetry code: (i) 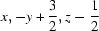.

**Fig. 1.**
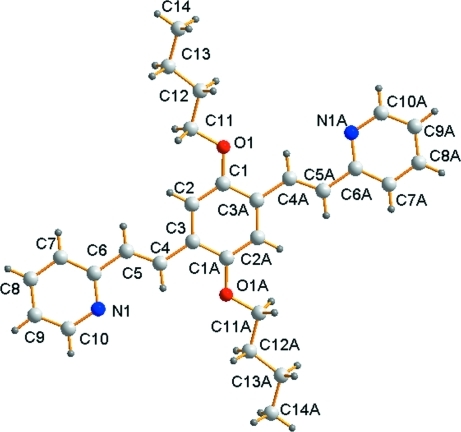
The molecular structure of the title molecule.

**Fig. 2.**
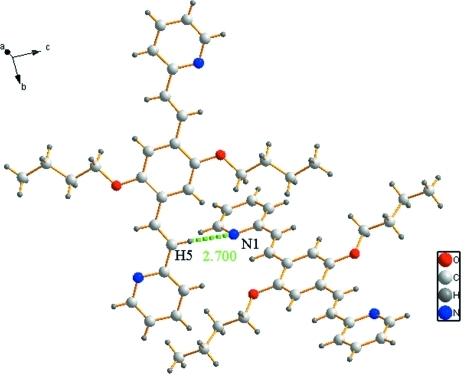
A view of the intermolecular C-H···N interactions (dashed lines) in the crystal structure of the title compound.

## supplementary crystallographic information

### Comment

Pyridine based materials have been investigated for their electrical and optical properties. The introduction of substituents in the 2- and 4-positions of pyridine represents a possible approach for designing pyridine-based photo-refractive materials (Naumov *et al.*, 2002; Liu *et al.*, 2008).

In the title molecule (Fig. 1), which is centrosymmetric, there are two pyridine rings and a central benzene ring. The dihedral angle between the pyridine and the central benzene ring is 11.29 (10) °.

In the crystal structure of the title compound there exist C5—H5···N1^i^ interactions between neighboring molecules [see Fig. 2 and Table 1]. This leads to the formation of a two-dimensional network lieing parallel to the *bc*-plane.

### Experimental

The title compound was prepared by firstly placing t-BuOK (8.98 g, 0.080 mol) in a dry motar and milling it to give very small particles. Then 2,5-Dibutoxy-1,4-bis(triphenylphosphonium)benzene dischloride (8.45 g, 0.010 mol) and picolinaldehyde (4.28 g, 0.040 mol) were added. The mixture was then milled vigorously for about 10 min. After the reaction was completed (monitored by TLC), the mixture was dispersed in 50 ml of H_2_O. The solution was extracted three times with 50 ml dichloromethane. The dichloromethane solution was dried for 12 h and concentrated. The concentrated solution was passed over a silica gel column and eluted with peroleum ether/ethyl acetate (8:1). By slow evaporation of the solvent yellow block-like crystals were obtained in 75% yield.

### Refinement

The H-atoms were placed in geometrically idealized positions and constrained to ride on their parent atoms: C—H = 0.93, 0.96 and 0.97 Å for CH, CH_3_ and CH_2_ H-atoms, respectively, with U_iso_(H) = k × U_eq_(parent C-atom), where k = 1.2 for CH and CH_2_ H-atoms and = 1.5 for CH_3_ H-atoms.

### Crystal data

**Table:**

C_28_H_32_N_2_O_2_	*F*(000) = 460
*M**_r_* = 428.56	*D*_x_ = 1.164 Mg m^−^^3^
Monoclinic, *P*2_1_/*c*	Mo *K*α radiation, λ = 0.71069 Å
Hall symbol: -P 2ybc	Cell parameters from 1446 reflections
*a* = 8.882 (5) Å	θ = 2.5–21.8°
*b* = 13.892 (5) Å	µ = 0.07 mm^−^^1^
*c* = 10.387 (5) Å	*T* = 298 K
β = 107.392 (5)°	Block, yellow
*V* = 1223.0 (10) Å^3^	0.50 × 0.30 × 0.20 mm
*Z* = 2	

### Data collection

**Table:**

Bruker SMART CCD area-detector diffractometer	2162 independent reflections
Radiation source: fine-focus sealed tube	1385 reflections with *I* > 2σ(*I*)
graphite	*R*_int_ = 0.040
phi and ω scans	θ_max_ = 25.0°, θ_min_ = 2.4°
Absorption correction: multi-scan (*SADABS*; Sheldrick, 1996)	*h* = −10→10
*T*_min_ = 0.964, *T*_max_ = 0.986	*k* = −16→16
8512 measured reflections	*l* = −11→12

### Refinement

**Table:**

Refinement on *F*^2^	Primary atom site location: structure-invariant direct methods
Least-squares matrix: full	Secondary atom site location: difference Fourier map
*R*[*F*^2^ > 2σ(*F*^2^)] = 0.049	Hydrogen site location: inferred from neighbouring sites
*wR*(*F*^2^) = 0.154	H-atom parameters constrained
*S* = 1.03	*w* = 1/[σ^2^(*F*_o_^2^) + (0.0821*P*)^2^ + 0.0525*P*] where *P* = (*F*_o_^2^ + 2*F*_c_^2^)/3
2162 reflections	(Δ/σ)_max_ < 0.001
146 parameters	Δρ_max_ = 0.16 e Å^−^^3^
0 restraints	Δρ_min_ = −0.16 e Å^−^^3^

### Special details

**Table:**

Geometry. All esds (except the esd in the dihedral angle between two l.s. planes) are estimated using the full covariance matrix. The cell esds are taken into account individually in the estimation of esds in distances, angles and torsion angles; correlations between esds in cell parameters are only used when they are defined by crystal symmetry. An approximate (isotropic) treatment of cell esds is used for estimating esds involving l.s. planes.
Refinement. Refinement of *F*^2^ against ALL reflections. The weighted *R*-factor wR and goodness of fit *S* are based on *F*^2^, conventional *R*-factors *R* are based on *F*, with *F* set to zero for negative *F*^2^. The threshold expression of *F*^2^ > σ(*F*^2^) is used only for calculating *R*-factors(gt) etc. and is not relevant to the choice of reflections for refinement. *R*-factors based on *F*^2^ are statistically about twice as large as those based on *F*, and *R*- factors based on ALL data will be even larger.

### Fractional atomic coordinates and isotropic or equivalent isotropic displacement parameters (Å^2^)

**Table:**

	*x*	*y*	*z*	*U*_iso_*/*U*_eq_	
O1	−0.10160 (17)	0.50961 (9)	0.22161 (12)	0.0589 (4)	
C2	0.0431 (2)	0.57863 (13)	0.43825 (17)	0.0477 (5)	
H2	0.0725	0.6313	0.3960	0.057*	
C1	−0.0472 (2)	0.50707 (13)	0.36022 (17)	0.0458 (5)	
C5	0.2548 (2)	0.72369 (13)	0.61936 (18)	0.0491 (5)	
H5	0.2324	0.7310	0.5265	0.059*	
C4	0.1898 (2)	0.64922 (13)	0.66286 (18)	0.0487 (5)	
H4	0.2083	0.6442	0.7556	0.058*	
C6	0.3587 (2)	0.79507 (14)	0.70512 (19)	0.0500 (5)	
C3	0.0919 (2)	0.57414 (13)	0.57915 (17)	0.0447 (5)	
N1	0.3841 (2)	0.78919 (14)	0.83829 (17)	0.0781 (6)	
C7	0.4305 (3)	0.86549 (15)	0.6486 (2)	0.0617 (6)	
H7	0.4106	0.8689	0.5556	0.074*	
C11	−0.0751 (3)	0.59382 (15)	0.15295 (19)	0.0620 (6)	
H11A	0.0359	0.5996	0.1601	0.074*	
H11B	−0.1069	0.6507	0.1922	0.074*	
C12	−0.1717 (3)	0.58404 (16)	0.00746 (18)	0.0639 (6)	
H12A	−0.1503	0.5217	−0.0253	0.077*	
H12B	−0.2826	0.5857	0.0020	0.077*	
C8	0.5312 (3)	0.93013 (17)	0.7318 (3)	0.0770 (7)	
H8	0.5797	0.9780	0.6957	0.092*	
C10	0.4832 (3)	0.8533 (2)	0.9145 (2)	0.0988 (10)	
H10	0.5018	0.8498	1.0074	0.119*	
C9	0.5588 (3)	0.9232 (2)	0.8671 (3)	0.0907 (9)	
H9	0.6277	0.9652	0.9258	0.109*	
C13	−0.1405 (4)	0.6606 (2)	−0.0831 (2)	0.0963 (9)	
H13A	−0.0302	0.6582	−0.0795	0.116*	
H13B	−0.1603	0.7231	−0.0499	0.116*	
C14	−0.2407 (4)	0.6502 (2)	−0.2276 (2)	0.0996 (10)	
H14A	−0.2273	0.5868	−0.2594	0.149*	
H14B	−0.2092	0.6972	−0.2822	0.149*	
H14C	−0.3496	0.6599	−0.2335	0.149*	

### Atomic displacement parameters (Å^2^)

**Table:**

	*U*^11^	*U*^22^	*U*^33^	*U*^12^	*U*^13^	*U*^23^
O1	0.0777 (10)	0.0571 (9)	0.0353 (8)	−0.0123 (7)	0.0069 (7)	−0.0006 (6)
C2	0.0541 (12)	0.0462 (10)	0.0408 (11)	−0.0014 (9)	0.0111 (9)	−0.0001 (8)
C1	0.0492 (11)	0.0514 (11)	0.0330 (10)	0.0032 (9)	0.0066 (9)	−0.0017 (8)
C5	0.0514 (12)	0.0543 (12)	0.0386 (11)	0.0007 (9)	0.0090 (9)	−0.0031 (9)
C4	0.0550 (12)	0.0529 (11)	0.0346 (11)	−0.0021 (9)	0.0081 (9)	−0.0050 (8)
C6	0.0510 (12)	0.0529 (12)	0.0467 (12)	−0.0029 (9)	0.0155 (10)	−0.0073 (9)
C3	0.0466 (11)	0.0463 (11)	0.0387 (10)	0.0006 (8)	0.0090 (9)	−0.0034 (8)
N1	0.0928 (15)	0.0965 (15)	0.0456 (11)	−0.0454 (12)	0.0215 (10)	−0.0188 (10)
C7	0.0672 (14)	0.0618 (13)	0.0568 (13)	−0.0111 (11)	0.0194 (11)	−0.0026 (10)
C11	0.0807 (16)	0.0550 (13)	0.0478 (12)	−0.0075 (11)	0.0154 (11)	0.0028 (10)
C12	0.0770 (16)	0.0679 (14)	0.0421 (12)	−0.0073 (11)	0.0107 (11)	0.0048 (10)
C8	0.0811 (17)	0.0685 (15)	0.0898 (19)	−0.0262 (13)	0.0385 (14)	−0.0114 (13)
C10	0.115 (2)	0.129 (2)	0.0536 (15)	−0.067 (2)	0.0270 (15)	−0.0310 (15)
C9	0.0903 (19)	0.105 (2)	0.0823 (19)	−0.0484 (16)	0.0338 (15)	−0.0386 (16)
C13	0.131 (2)	0.0873 (18)	0.0572 (16)	−0.0271 (17)	0.0078 (16)	0.0158 (13)
C14	0.125 (3)	0.108 (2)	0.0532 (15)	−0.0132 (18)	0.0064 (15)	0.0242 (14)

### Geometric parameters (Å, °)

**Table:**

O1—C1	1.375 (2)	C11—C12	1.504 (3)
O1—C11	1.426 (2)	C11—H11A	0.9700
C2—C1	1.379 (3)	C11—H11B	0.9700
C2—C3	1.398 (2)	C12—C13	1.499 (3)
C2—H2	0.9300	C12—H12A	0.9700
C1—C3^i^	1.406 (3)	C12—H12B	0.9700
C5—C4	1.328 (3)	C8—C9	1.356 (3)
C5—C6	1.462 (3)	C8—H8	0.9300
C5—H5	0.9300	C10—C9	1.353 (3)
C4—C3	1.466 (3)	C10—H10	0.9300
C4—H4	0.9300	C9—H9	0.9300
C6—N1	1.336 (2)	C13—C14	1.506 (3)
C6—C7	1.390 (3)	C13—H13A	0.9700
C3—C1^i^	1.406 (3)	C13—H13B	0.9700
N1—C10	1.334 (3)	C14—H14A	0.9600
C7—C8	1.375 (3)	C14—H14B	0.9600
C7—H7	0.9300	C14—H14C	0.9600
			
C1—O1—C11	119.07 (14)	H11A—C11—H11B	108.5
C1—C2—C3	121.80 (18)	C13—C12—C11	114.19 (19)
C1—C2—H2	119.1	C13—C12—H12A	108.7
C3—C2—H2	119.1	C11—C12—H12A	108.7
O1—C1—C2	123.88 (17)	C13—C12—H12B	108.7
O1—C1—C3^i^	115.58 (15)	C11—C12—H12B	108.7
C2—C1—C3^i^	120.53 (17)	H12A—C12—H12B	107.6
C4—C5—C6	125.48 (18)	C9—C8—C7	119.1 (2)
C4—C5—H5	117.3	C9—C8—H8	120.4
C6—C5—H5	117.3	C7—C8—H8	120.4
C5—C4—C3	126.43 (18)	N1—C10—C9	125.0 (2)
C5—C4—H4	116.8	N1—C10—H10	117.5
C3—C4—H4	116.8	C9—C10—H10	117.5
N1—C6—C7	121.57 (18)	C10—C9—C8	118.2 (2)
N1—C6—C5	118.03 (18)	C10—C9—H9	120.9
C7—C6—C5	120.38 (18)	C8—C9—H9	120.9
C2—C3—C1^i^	117.66 (16)	C12—C13—C14	113.1 (2)
C2—C3—C4	122.17 (17)	C12—C13—H13A	109.0
C1^i^—C3—C4	120.15 (16)	C14—C13—H13A	109.0
C10—N1—C6	116.8 (2)	C12—C13—H13B	109.0
C8—C7—C6	119.3 (2)	C14—C13—H13B	109.0
C8—C7—H7	120.4	H13A—C13—H13B	107.8
C6—C7—H7	120.4	C13—C14—H14A	109.5
O1—C11—C12	107.37 (16)	C13—C14—H14B	109.5
O1—C11—H11A	110.2	H14A—C14—H14B	109.5
C12—C11—H11A	110.2	C13—C14—H14C	109.5
O1—C11—H11B	110.2	H14A—C14—H14C	109.5
C12—C11—H11B	110.2	H14B—C14—H14C	109.5

Symmetry codes: (i) −*x*, −*y*+1, −*z*+1.

### Hydrogen-bond geometry (Å, °)

**Table:**

*D*—H···*A*	*D*—H	H···*A*	*D*···*A*	*D*—H···*A*
C5—H5···N1^ii^	0.93	2.70	3.446 (3)	138

Symmetry codes: (ii) *x*, −*y*+3/2, *z*−1/2.
